# TM4SF1-Directed Antibody–Drug Conjugates Selectively Destroy Newly Formed Blood Vessels Induced by VEGF-A

**DOI:** 10.3390/ijms27104437

**Published:** 2026-05-15

**Authors:** Chi-Iou Lin, Yu Liu, Tracey E. Sciuto, Anne Merley, Harold F. Dvorak, Shou-Ching S. Jaminet

**Affiliations:** 1 Center for Vascular Biology Research, Department of Pathology, Beth Israel Deaconess Medical Center, Harvard Medical School, Boston, MA 02130, USA; 2 Riverview Hospital, 395 Westfield Rd., Noblesville, IN 46060, USA; 3 Department of Pharmacology, Shanxi Medical University, Xinjiannanlu 56, Taiyuan 030001, China; 4 Center for Animal Resources and Education, Brown University, Providence, RI 02912, USA; 5 Angiex Inc., Cambridge, MA 02140, USA

**Keywords:** TM4SF1, ADC, VEGF-A, angiogenesis, anti-angiogenic therapy

## Abstract

Antibody–drug conjugates (ADCs) are a promising therapeutic modality for treating cancer. TM4SF1 is an integral membrane protein that internalizes from the cell surface along microtubules to the nucleus and is highly expressed on the surface of both tumor endothelium and tumor cells. We previously reported that in human tumor xenografts in mice, an ADC directed to mouse TM4SF1 (2A7A-LP2) effectively regressed tumors through an anti-vascular mechanism, and an ADC directed to human TM4SF1 (v1.10-LP2) effectively regressed tumors through an anti-tumor cell mechanism. In this study, we investigated the actions of the mouse TM4SF1-directed ADC on VEGF-A-provoked angiogenic vessels. We employed an adenovirus expressing mouse VEGF-A^164^ (Ad-VEGF-A) to induce surrogate tumor blood vessels in the ears of nude mice. We showed that an immune effector function-ablated ADC, 3m2A7A-LP2, was better tolerated than its parent 2A7A-LP2. Homing of 3m2A7A to Ad-VEGF-A-induced new blood vessels became evident within six hours after intraperitoneal injection. A single dose of 3m2A7A-LP2 at 3 mg/kg disrupted evolving Ad-VEGF-A-provoked blood vessels within forty-eight hours, and three doses of 3m2A7A-LP2 at 48 h intervals caused striking local ear necrosis; in each case, there was no apparent harm to vessels in the corresponding control virus-injected ears and the surrounding tissues of the same mice. Our studies demonstrate that an ADC-directed against mouse TM4SF1 specifically targeted the newly formed blood vessels induced by Ad-VEGF-A at multiple stages of their development. Thus, TM4SF1-directed ADCs, through their ability to target angiogenic vessels, represent an alternative anti-angiogenic approach for treating solid tumors.

## 1. Introduction

Blood vessels supplying solid tumors are heterogeneous and can be classified into at least six distinctly different types: mother vessels, glomeruloid microvascular proliferations, capillaries, vascular malformations, feeding arteries, and draining veins [1,2]. These different vessel types can be sequentially recapitulated in nude mouse ear skin in the absence of tumor cells with an adenoviral vector engineered to express murine vascular endothelial growth factor-A (Ad-VEGF-A) [1]. This tumor-surrogate angiogenesis model has been exploited to assess the therapeutic potential of anti-angiogenic agents that block VEGF-A or its receptor VEGF-R [3,4]. Examples include “VEGF traps” (e.g., Eylea^®^), antibodies directed against VEGF-A (e.g., Avastin^®^) or VEGF-R (e.g., Cyramza^®^), and VEGF-R tyrosine kinase inhibitors (e.g., Sutent^®^) [1,3,4]. Therapeutic treatment of cancers with these agents had limited clinical benefit as a monotherapy but in some instances delayed tumor recurrence when combined with chemotherapy [5,6]. Therefore, if anti-vascular therapy is to become more effective, additional targets beyond the VEGF-VEGFR axis are required [3].

Transmembrane-4-L-Six Family member-1 (TM4SF1) is an integral plasma membrane glycoprotein that was discovered in 1986 as a tumor cell-associated antigen [7]. We subsequently identified TM4SF1 as a vascular endothelial cell biomarker whose expression is uniformly high in cultured endothelial cells of various origins, in vivo on human tumor vascular endothelium [8], pathological angiogenic blood vessels induced in mice in retinopathy of prematurity [9], and Ad-VEGF-A-provoked angiogenesis [8]. When highly expressed, TM4SF1 forms multiple 100–300 nm diameter clusters on the surface of cultured endothelial and tumor cells [10]. These clusters, referred to as TM4SF1-enriched microdomains (TMEDs), include a variety of additional proteins that TM4SF1 recruits and that together internalize from the cell surface along microtubules to the nucleus [11]. In this manner, TM4SF1-recruited proteins can be distributed into intracellular compartments and are essential for blood vessel development [10].

TM4SF1 has emerged as an attractive antibody–drug conjugate (ADC) target because of (a) its potential to dually target both tumor cells and the tumor vasculature [12], and (b) its unique internalization pathway to the nucleus [13], independent of the endosomes or lysosomes by which many other surface molecules enter cells to deliver conjugated payloads [13,14]. In collaboration with Pfizer Corp., we developed the first TM4SF1-directed ADCs specifically against human (v1.10-LP2) and mouse (2A7A-LP2) TM4SF1 [12]. Both were highly effective as single agents against tumor xenografts, and still more effective when combined for dual targeting of human tumor cells via v1.10-LP2 and mouse tumor vasculature via 2A7A-LP2 [12].

In this study, we examined the anti-angiogenic activities of 2A7A-LP2 in the VEGF-A-induced surrogate tumor blood vessels model. We report that 3m2A7A, an antibody derived from 2A7A by introduction of the L234A-L235A-G237A mutations to diminish FcγR binding [15,16], efficiently homed to Ad-VEGF-A-induced blood vessels. 3m2A7A-LP2, the ADC formed by conjugating 3m2A7A to a microtubule toxin, was better tolerated than its parent molecule, 2A7A-LP2, and effectively targeted newly evolving blood vessels from Ad-VEGF-A expression with no apparent effect on the surrounding non-angiogenic normal blood vessels or the tissues that they supplied. These studies reveal that TM4SF1-directed ADCs can be effective anti-angiogenic agents and provide a novel and attractive approach for treating solid tumors. Currently, a TM4SF1-directed ADC developed by Angiex, Inc. is in Phase 1 clinical trials.

## 2. Results

### 2.1. Cytotoxicity and Toxicity of TM4SF1-Directed ADC 3m2A7A-LP2 and Its Parent Molecule 2A7A-LP2

The linker/payload LP2 comprises a maleimidocaproyl linker that is attached to a synthetic dolastatin-10 analog (PF-06463377; the derivatives of taxanes with the structure resemble momomethyl auristatin-F MMAF) via an amide bond [17]. MMAF is a microtubule-destabilizing agent that inhibits tubulin polymerization [18] and induces cell cycle arrest at the G2/M phase, ultimately leading to apoptosis in rapidly dividing cells [19,20].

**Figure 1:**
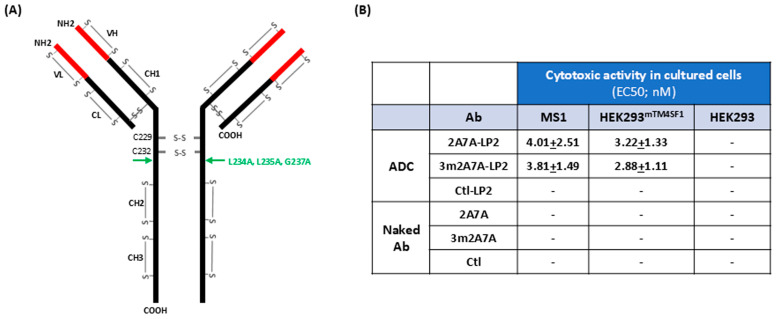
Cytotoxicity of TM4SF1-directed ADC 3m2A7A-LP2 and its parent molecule 2A7A-LP2. (**A**) Human IgG1 2D structure with the site of the “LALAGA” effector function ablating mutations. The illustration depicts the various intra- and inter-chain disulfide bonds that connect heavy and light chains of the human IgG1 antibody, and their positions relative to the three amino acid mutations (3m: L234A, L235A, and G237A; green fonts and arrow) known to diminish IgG1 interactions with Fcγ receptors. L, leucine; A, alanine; G, glycine. The linker/payload LP2 (mc-3377) was randomly conjugated to the antibody via lysine residue, with an average drug-to-antibody ratio of four. Three ADCs (2A7A-LP2, 3m2A7A-LP2, and Ctl-LP2) with an average drug-to-antibody ratio of four were generated by randomly conjugating the LP2 to cysteine residues. (**B**) Cytotoxic activities. Cell viability was assessed at Day 5 through PrestoBlue assays to evaluate mouse TM4SF1-directed ADCs and their respective naked antibodies against cultured MS1 (immortalized mouse endothelial cell that expresses high levels of TM4SF1), HEK293 (TM4SF1 null cells), and HEK293^mTM4SF1^ (HEK293 stably transduced to express mouse TM4SF1) cells in vitro. Both anti-mouse TM4SF1 ADCs, 2A7A-LP2 and 3m2A7A-LP2, delivered comparable cytotoxic activities against MS1 and HEK293^mTM4SF1^ cells with EC50 of 2–4 nM. The isotype-matched control ADC, Ctl-LP2, and the naked antibodies of all three ADCs did not show detectable cytotoxic activity in either cell type. -: no cytotoxicity.

In vitro, the effector function null ADC 3m2A7A-LP2 showed comparable cytotoxic activity in comparison to its parent ADC 2A7A-LP2 against cultured mouse endothelial cell MS1 and mouse TM4SF1 stably transformed HEK293 cells (Figure 1; EC50 = 2–4 nM).

**Figure 2:**
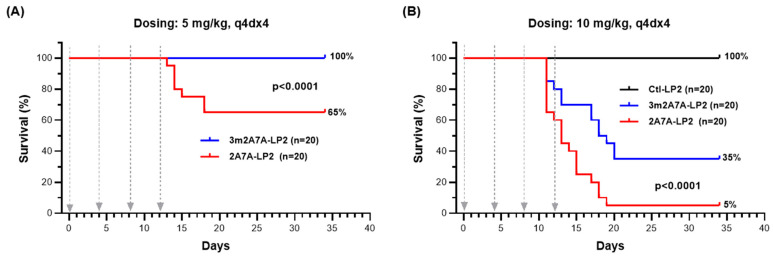
Toxicity of ADCs directed against mouse TM4SF1 in C57BL/6 mice. Kaplan–Meier survival curves of eight-week-old C57BL/6 female mice that received intraperitoneal injections of 2A7A-LP2, 3m2A7A-LP2 or control ADC Ctl-LP2 at 5 mg/kg (**A**) or 10 mg/kg (**B**), with q4dx4 dosing schedules (gray arrows). Each study group contained 10 mice and was repeated twice in side-by-side experiments for all three ADCs, totaling *n* = 20/group. 3m2A7A-LP2 had a 100% and 35% survival rate respectively at 5 mg/kg and 10 mg/kg (*p* < 0.0001), while 2A7A-LP2 achieved 65% and 5% survival respectively at 5 mg/kg and 10 mg/kg (*p* < 0.0001). Ctl-LP2 showed 100% survival at 10 mg/kg. Log-rank test for *p*-value calculations.

In vivo, nude mice were subjected to q4dx4 (four-day intervals for four cycles) intraperitoneal (ip) treatments with 2A7A-LP2 or 3m2A7A-LP2. Kaplan–Meier survival curves from mice treated with the 5 mg/kg dose showed 65% and 100% survival after treatments with 2A7A-LP2 and 3m2A7A-LP2, respectively, over a 34-day study period (Figure 2A; *p* < 0.0001, log-rank test). Higher dose levels (10 mg/kg, q4dx4) of these ADCs were more toxic: 5% and 35% survival for 2A7A-LP2 and 3m2A7A-LP2 treated mice, respectively (Figure 2B; *p* < 0.0001, log-rank test). Mice receiving isotype-matched control Ctl-LP2 demonstrated no evidence of toxicity, and survival was 100% at both 5 mg/kg) and 10 mg/kg doses (Figure 2B) following the same dosing regimens.

### 2.2. Blood Vessel Homing of 3m2A7A Antibody

**Figure 3:**
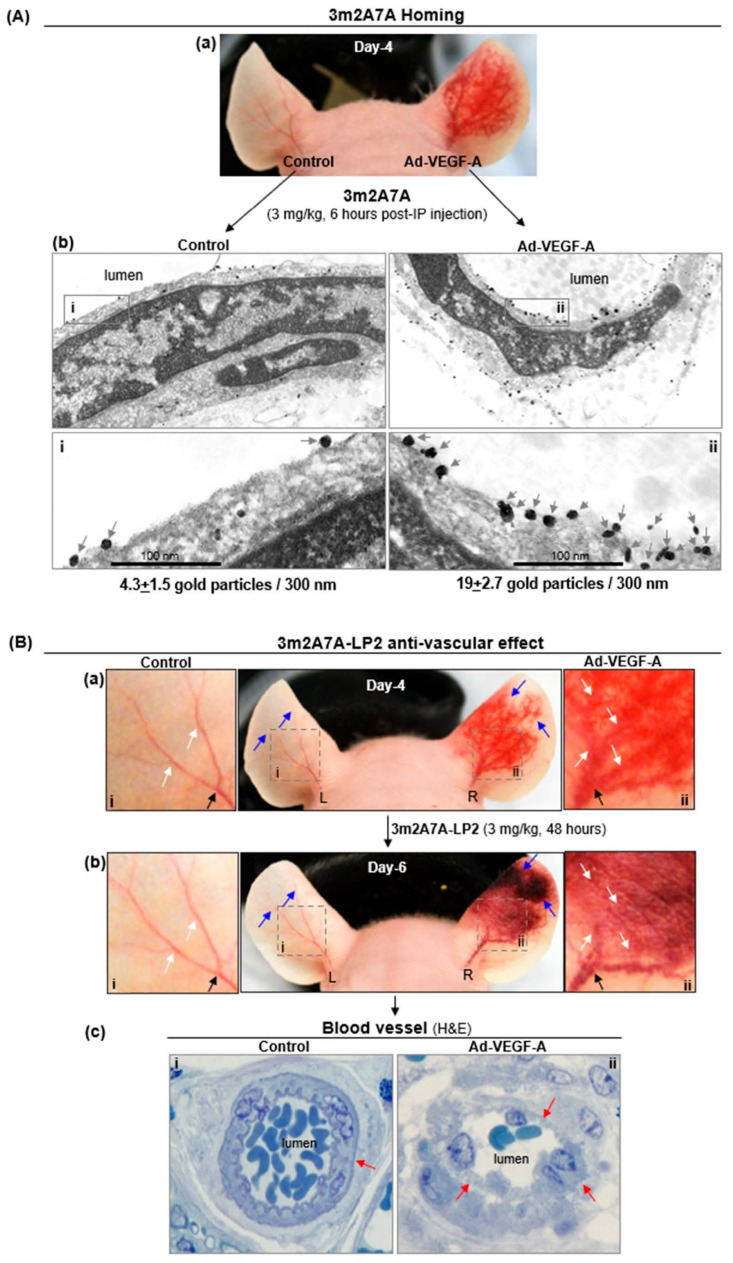
Preferential homing of 3m2A7A and 3m2A7A-LP to microvasculature in Ad-VEGF-injected sites. Nude mice received intradermal ear injections of 2.5 × 10^7^ pfu mock control (Control, left-ear L) or mouse VEGF-A^164^ (Ad-VEGF-A, right-ear R) adenovirus. Four days later (**Aa**,**Ba**), mice received a single 3 mg/kg intraperitoneal injection of 3m2A7A (**A**) or 3m2A7A-LP2 (**B**). Mouse ears were harvested for immuno-electron microscopy 6 h later (**A**) or for histology 48 h later (**B**). Nanogold-labeled anti-human IgGs were used to identify 3m2A7A (**Ab**). Representative images from three different ears of three different mice show few gold particles (indicated by gray arrows in inset **i** and **ii**) on microvascular endothelial luminal surface of the normal control ear (Control; (**Ab**(**i**)), three gold particles per 300 nm plasma membrane), but numerous dense clusters on the endothelial luminal surface of the Ad-VEGF ear (Ad-VEGF-A; (**Ab**(**ii**)), eighteen gold particles per 300 nm plasma membrane); the mean ± SD of gold particles in a representative 300 nm membrane section from control and Ad-VEGF-A-treated ears was 4.3 ± 1.5 and 19 ± 2.7 respectively (3 ears per group; a 4.42-fold difference; *p* = 0.002, Student’s *t*-test). (**B**) Representative images show vascular damage to the angiogenic vessels of Ad-VEGF-A-injected ears but not to mock control ears (compare the blue and white arrows in (**Ba**,**Bb**) and inset **i**, **ii**; black arrows indicate feeding arteries). (**Bc**) Light microscopic images show damaged vascular endothelium in the Ad-VEGF-A-injected angiogenic ear but not in the control ear (red arrows).

An adenoviral vector expressing mouse VEGF-A164 (Ad-VEGF-A) and a mock control adenovirus were respectively injected into the right and left ears of nude mice. As expected, Ad-VEGF-A, but not the control virus, induced a strong angiogenic response (Figure 3Aa) [1]. Four days after the viral injection, 3m2A7A (3 mg/kg) was injected intraperitoneally. Then, six hours later, mouse ears were harvested for electron microscopy to identify ear microvessel-bound 3m2A7A antibody, using nanogold-labeled anti-human IgGs. Microvessel endothelial cells at Ad-VEGF-A-injected sites exhibited numerous clusters of nanogold particles bound to their luminal surface, whereas in control ears, endothelial cells exhibited only a few scattered nanogold particles; a 4.42-fold higher level of gold particles was quantified in Ad-VEGF-A ears than in control ears (Figure 3Ab). An isotype-matched control antibody (Ctl) showed no interaction with the microvascular endothelium from the Ad-VEGF-A-injected ear (Supplementary Figure S1A).

### 2.3. 3m2A7A-LP2 Selectively Damaged Newly Formed Ad-VEGF-A-Induced Blood Vessels

Ad-VEGF-A and control adenoviruses were injected into nude mouse ears in Figure 3A. Four days later (Day 4), mice were injected ip with a single 3 mg/kg dose of 3m2A7A-LP2 (Figure 3Ba) or the Ctl-LP2 control ADC (Supplementary Figure S1). Two days thereafter (Day 6), striking blood vessel damage was observed both macroscopically (Figure 3Bb) and microscopically (Figure 3Bc) in the newly formed blood vessels of Ad-VEGF-A-injected ears of mice treated with 3m2A7A-LP2, whereas blood vessels in mock control-injected ears appeared normal in the same mice. Vessels in the ears of mice treated with Ctl-LP2 also remained intact, regardless of whether the ear had been injected with Ad-VEGF-A or mock control (Supplementary Figure S1).

### 2.4. Plasma Albumin Content in Ad-VEGF-A- and Control-Injected Ears with 3m2A7A-LP2 or Ctl-LP2 Treatment

**Figure 4:**
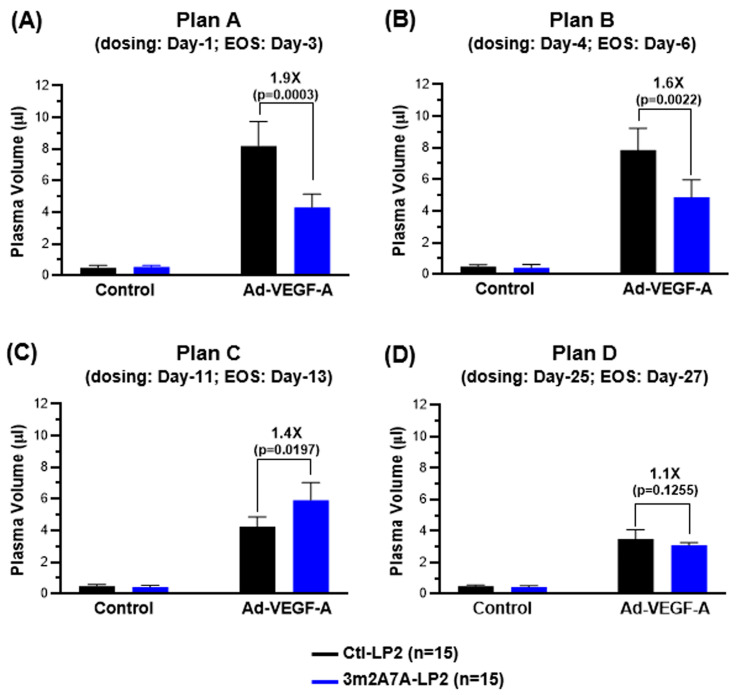
Targeting VEGF-A-provoked angiogenic blood vessels with 3m2A7A-LP2. Mice were divided into two groups of five mice each after intradermal injections of adenoviruses as described in Figure 2; they received a single intraperitoneal injection of either 3 mg/kg 3m2A7A-LP2 or Ctl-LP2 at (**A**) Day-1 (Plan A), (**B**) Day-4 (Plan B), (**C**) Day-11 (Plan C), or (**D**) Day-25 (Plan D). Evans blue dye (EBD) was injected retro-orbitally at the end of the study (EOS), and the sites of adenovirus injections were harvested via 8 mm biopsy punches for EBD extraction to quantify plasma volumes. Studies were repeated three times, and plasma volumes (*n* = 15, mean ± SD) are shown. The differences in plasma volume accumulations between 3m2A7A-LP2 and Ctl-LP2 treatments in Plans A–D were, respectively, 1.9-fold higher (*p* = 0.0003), 1.6-fold higher (*p* = 0.0022), 1.4-fold lower (*p* = 0.0197), and equivalent (1.1-fold, *p* = 0.1255). Student’s *t*-test for *p*-value calculations.

We have previously described the evolution of the angiogenic response induced by Ad-VEGF-A in nude mouse ears [1]. Ad-VEGF-A induces a strong angiogenic response within days, which then recedes over the course of several weeks (Supplementary Figure S2); in contrast, control virus-injected sites appear normal at all times (Figure 4, left years). We employed Evans blue dye (EBD) to quantify these changes, as well as the effects of 3m2A7A-LP2 and Ctl-LP2 on the vasculature. When injected iv, EBD binds to plasma albumin and thus provides a measure of net plasma albumin accumulation, including both that within the vasculature and any that has leaked out [4].

At different times after Ad-VEGF-A and mock control adenovirus injection into the right and left ears of nude mouse ears (four experimental Plans A–D; Supplementary Figure S2, Column C), we treated mice with a single ip dose of either 3m2A7A-LP2 or Ctl-LP2; two days thereafter, mice were injected iv with EBD and ten minutes later, injected ear sites were biopsied with an 8 mm punch for EBD extraction. The results are shown in Figure 4 (Plans A–D). As expected, accumulation of EBD was low and equivalent in all four experimental Plans in the ears of nude mice injected with control virus, whether or not followed by 3m2A7A-LP2 or Ctl-LP2 treatments. In contrast, EBD accumulation was greatly increased in nude mice whose ears had been injected with Ad-VEGF-A, followed by treatment with a single ip injection of Ctl-LP2 ADC in all four Plans; accumulations paralleled the increasing and waning angiogenic response, i.e., exhibiting maximal accumulations at days 3 and 6 (Plans A and B), with lower but still significantly increased accumulations at later times (Plans C and D), as the new vasculature receded and stabilized. In contrast, 3m2A7A-LP2 very significantly reduced ear EBD content in Ad-VEGF-A-injected ears in Plans A (days 1–3, *p* = 0.0003) and Plan B (days 4–6, *p* = 0.0022). However, in Plan C (days 13–15), EBD content in 3m2A7A-LP2-treated mice was somewhat greater than in control ears (*p* = 0.0197). In Plan D (days 27–29), EBD accumulation did not differ significantly from that of Ctl-LP2-treated mice, as the angiogenic response, while still evident, was on the wane, and the newly formed vasculature had been pruned or had matured and was no longer susceptible to 3m2A7A-LP2.

### 2.5. Macroscopic Appearance of Nude Mouse Ears at Different Times After Ad-VEGF-A Adenovirus Injection, Followed by ADC or Respective Naked Antibody Treatments

**Figure 5:**
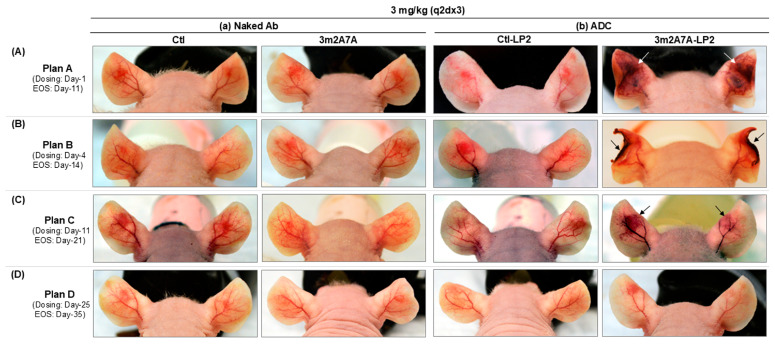
Effects of 3m2A7A-LP2, 3m2A7A, Ctl and Ctl-LP2 on ear sites at various times after Ad-VEGF-A injection. Both ears of nude mice received 2.5 × 10^7^ pfu Ad-VEGF-A on Day 0. Mice were then divided into four groups (5 mice/group; corresponding to experimental Plans A–D as in Figure 4) and received three cycles of 3 mg/kg of indicated (**a**) naked antibodies or (**b**) their respective ADCs ip at two-day intervals, beginning at the indicated times. Ears were photographed at the end of the study (EOS), the tenth day after initial dosing in each experimental plan. 3mA7a-LP2 caused severe damage to the vasculature at Ad-VEGF-A-injected sites in (**A**) Plan A and (**B**) Plan B (note especially ear necrosis in Plan B), while causing less damage in (**C**) Plan C, or no (**D**) in Plan D. In contrast, treatments with Ctl, 3mA7A and Ctl-LP2 caused no ear damage in any of the plans. For further details, see Supplementary Figure S3.

Finally, we injected Ad-VEGF-A into both left and right ears of nude mice, and beginning on days-1, -4, -11, or -25 (according to the four experimental Plans A–D, Figure 5), administered a course of ip injections (q2dx3) of 3 mg/kg ADCs (3m2A7A-LP2 or Ctl-LP2) or their respective naked antibodies (3m2A7A or Ctl) without the mc-3377 payload. Representative ear images taken at the end of the study (EOS), the tenth day from the initial dosing, demonstrate the effects of these antibody treatments (Figure 5). Naked 3m2A7A and Ctl antibodies, as well as Ctl-LP2, had no demonstrable effect on the ear vasculature in any of the four Plans. In contrast, 3m2A7A-LP2 treatment extensively damaged ear blood vessels, leading to tissue necrosis by the tenth day after the start of dosing in Plans A (Figure 4A) and B (Figure 4B). 3m2A7A-LP2 had a lesser effect on blood vessels when administered at later times (days 11–21, Plan C; Figure 4C) and no discernible effect in mice treated on days 25–35 (Plan D; Figure 4D) when the angiogenic response had waned and remaining new blood vessels had matured. Sequential ear images taken in response to the different treatments are presented in Supplementary Figure S3.

## 3. Discussion

TM4SF1 is an endothelial cell biomarker whose expression is increased when endothelial cells are activated to proliferate and migrate [8,10]. This study reports that a mouse TM4SF1-directed antibody-drug conjugate (ADC) selectively targets the newly formed blood vessels induced by an adenovirus-expressing VEGF-A in mice in vivo. Like cultured endothelial cells or cells transformed to overexpress TM4SF1 (Figure 1B), murine endothelial cells lining the newly formed blood vessels induced by Ad-VEGF-A in vivo expressed high levels of TM4SF1 [8] and hence were vulnerable to the mouse TM4SF1-directed ADC (Figure 3, Figure 4 and Figure 5). Conversely, endothelial cells lining normal blood vessels expressed low levels of TM4SF1 and were spared from the TM4SF1-directed ADC (Figure 3, Figure 4 and Figure 5).

TM4SF1 expression levels correlate with a cell’s sensitivity to the TM4SF1-directed ADC, a correlation we originally noted from cultured tumor cells that express varying levels of TM4SF1 [12]. Electron microscopy with a nanogold-labeled antibody showed few TM4SF1 molecules on the surface of normal endothelium (Figure 3Ab(i)), and numerous clusters of TM4SF1 on angiogenic endothelium (Figure 3Ab(ii)). Similar observations were noted in previous studies comparing TM4SF1 expression on the endothelium of human cancers and adjacent normal specimens [11]. Apparently, TMEDs (TM4SF1-enriched microdomains) only form when TM4SF1 expression has surpassed a threshold. If so, then internalization of TMEDs via microtubules will be restricted to activated angiogenic endothelium; this would explain the insensitivity of normal endothelium to the TM4SF1-directed ADC. Specifically, the endothelium of the mock control ears, the treated ear surrounding the Ad-VEGF-A-affected area, and the normal blood vessels in the other areas of the body were resistant to the 3 mg/kg dosing of the ADC (Figure 3, Figure 4 and Figure 5).

Due to the expression of murine TM4SF1 on the vasculature, the ADC directed to mouse TM4SF1 was rapidly cleared in mice in vivo in comparison to control ADCs, including an ADC directed to human TM4SF1 [12]. Residual TM4SF1 expression on the normal endothelium may lead to unwanted immune-mediated toxicities through Fc effector functions of antibodies bound to TM4SF1 on the vascular wall. Such antibody-mediated toxicities may arise from antibody-dependent cellular cytotoxicity (ADCC) activity, antibody-dependent cellular phagocytosis (ADCP), and complement-dependent cytotoxicity (CDC) mediated through binding to Fc gamma receptors (FcγR) expressed on leukocytes and/or lymphocytes [15,21]. Alternatively, the TM4SF1-directed ADC may induce payload-mediated toxicity to vascular endothelial cells, as TM4SF1 can be selectively recruited to classic tetraspanin microdomains [10,22], which then enter cells via vesicular internalization with subsequent payload release in lysosomes to deliver cytotoxicity [23,24]. Mice tolerated 3m2A7A-LP2 better than its parent molecule, 2A7A-LP2, indicating that Fc effector functions, ablated in 3m2A7A to minimize ADCC, ADCP and CDC activities, contribute to toxicity (Figure 2B). Although payload-mediated toxicity could be observed at high doses of 3m2A7A-LP2 (such as a total amount of 40 mg/kg in twelve days injected on a q4dx4 schedule) (Figure 2), we have not noted such toxicity at 3 mg/kg q4dx4 or q2dx4 dosing schemes (12 mg/kg total). Here, we employed 3 mg/kg q2dx3 (9 mg/kg total) as the highest dose to examine efficacy against Ad-VEGF-A-provoked vessels.

Homing of 3m2A7A to four-day Ad-VEGF-A-induced angiogenic vessels was seen as early as six hours after ip injection (Figure 3Ab). 3m2A7A without payload had no cytotoxic effect on cultured endothelial cells (Figure 1B), or on the quiescent or VEGF-A-activated endothelium in vivo (Figure 5), independent of TM4SF1 expression levels. However, a single dose of the 3m2A7A ADC (3m2A7A-LP2) extensively damaged blood vessels at Ad-VEGF-A injection sites by forty-eight hours (Figure 3B), and three consecutive injections of 3 mg/kg 3m2A7A-LP2 at 48 h intervals caused severe vascular damage, resulting in ear sloughing at Ad-VEGF-A injection sites (Figure 5B).

It has been difficult to devise a means of quantifying the intensity of angiogenesis and of its reduction in response to therapy. We devised a possible method for doing so using Evans blue dye (EBD) [4,25,26]. Injected iv, EBD binds to plasma albumin and so provides a quantitative measure of plasma albumin accumulation in tissues, including that both within and outside the vasculature, at any given point of time [27]. As expected, little EBD was measured in ears that received mock-control adenovirus and Ctl-LP2 (Figure 4); the EBD measured in these normally appearing ears was primarily that within the vasculature, as these vessels exhibited no increase in vascular permeability to circulating proteins such as albumin. EBD accumulation was dramatically increased in ears exhibiting an angiogenic response induced by Ad-VEGF-A, where EBD accumulation paralleled the increased density and permeability status of the newly formed vasculature. Accumulation was maximal at early times after Ad-VEGF-A injection when vascular density and permeability were maximal (corresponding to a predominance of “mother vessels” as in Plans A and B), and decreased as newly formed blood vessels declined in number and permeability (coincident with the maturing of angiogenic vessels as in Plans C and D). Treatment with 3m2A7A-LP2 strikingly reduced EBD accumulation in the Ad-VEGF-A-injected ears of Plans A and B, reflecting vascular damage that restricted the amount of circulating EBD able to enter ear sites. Some degree of ear damage also occurred with 3m2A7A-LP2 at Day 11 dosing, though a visible effect required two doses, indicating that more mature vessels had become more resistant to the ADC (Figure 4C, Plan C; Supplementary Figure S3C). With 3m2A7A-LP2 dosing on days 25–35 (Figure 4 and Figure 5; Plan D), there was no longer a measurable effect on EBD tissue accumulation, indicating that vascular permeability was no longer being altered by the drug. Thus, with sufficient lapse of time from the Ad-VEGF-A injection, 3m2A7A-LP2 spared the Ad-VEGF-A-influenced vessels in the same manner that it spared normal vessels never exposed to Ad-VEGF-A.

Approved anti-angiogenic therapies, such as bevacizumab (Avastin^®^) or ramucirumab (Cyramza^®^), inhibit VEGF-A- or VEGF-R-mediated signaling, with the aim of reducing growth factor stimulation of endothelial cells and thereby inhibiting their proliferation for new blood vessel formation [28,29]. TM4SF1-directed ADCs, by contrast, deliver cytotoxins to endothelial cells and are intended to cause vascular injury and elimination of angiogenic blood vessels [12]. In the Ad-VEGF-A model, mother vessels and capillaries, which are preferentially targeted by therapies aimed at VEGF-A or its receptors [3,4], are also effectively eliminated by TM4SF1-directed ADC (Figure 3Bb; white arrows). However, vessels that are surrounded by mural cells, such as arterioles, are insensitive to currently approved angiogenic therapies [3,4], were also damaged by the TM4SF1-directed ADC (Figure 3Bc). Therefore, ADC therapies directed against TM4SF1 have the potential to provide more effective targeting of angiogenic vessels in solid tumors while retaining a wide margin of safety because normal quiescent endothelial cells and other cells that normally express low levels of TM4SF1 are spared (Figure 3, Figure 4 and Figure 5). Furthermore, clinical trials have shown that normal endothelium is generally insensitive to tubulin inhibitors (e.g., vinca alkaloids, auristatins, taxanes, tubulysin or combretastatins) [30,31], suggesting the suitability of tubulin inhibitor payloads for TM4SF1-directed ADCs. In sum, TM4SF1-directed ADC microtubule-inhibiting payloads hold potential to effectively target the vasculature of solid tumors with a good therapeutic margin. A TM4SF1-directed microtubule-targeting ADC, AGX101, is currently being tested in Phase 1 clinical trials by Angiex Inc. The trial has primary safety endpoints and secondary efficacy endpoints in an all-solid cancer patient population.

## 4. Materials and Methods (See Supplementary Materials and Methods for Additional Details)

### 4.1. Antibodies and Antibody–Drug Conjugates (ADCs)

Two rabbit anti-mouse TM4SF1 antibodies (2A7A and 3m2A7A, see Supplementary Figure S1) and a non-targeting isotype-matched control antibody 8.8 (Ctl), and their respective ADCs (2A7A-LP2, 3m2A7A-LP2 and Ctl-LP2) were employed [12]. All three antibodies have a human IgG1 constant region [12]. The linker/payload LP2 (mc-3377) comprises a maleimidocaproyl (mc) linker that is attached to a Pfizer-developed synthetic dolastatin-10 analog microtubule cytotoxin PF-06463377 (structure resembles momomethyl auristatin-F) via an amide bond, and randomly conjugated to lysine residues on the antibodies [17]. The payload is able to induce G2–M cell-cycle arrest and cytotoxicity became evident within 24–48 h after internalization [17]. Average LP2 drug-to-antibody ratio (DAR), as we have previously reported [12], was 4 for all of the ADCs used in this study. A nanogold-labeled goat anti-human IgG (Nanoprobes, Yaphank, NY, USA) was used as a secondary antibody in electron microscopy.

### 4.2. Cell Lines

The MS1, the immortalized mouse endothelial cell line, expresses high levels of TM4SF1 [12], was purchased from ATCC (Manassas, VA, USA) and maintained according to the vendor’s recommended conditions. HEK293^mTM4SF1^ (Human embryonic kidney 293) cells were stably transfected to express mouse TM4SF1 [12] and maintained in RPMI/10% FBS media and supplemented with sodium pyruvate and non-essential amino acids. MS1, HEK293 and HEK293^mTM4SF1^ cells were used for the evaluation of 2A7A-LP2 and 3m2A7A-LP2 cytotoxic activities in vitro [12].

### 4.3. Cytotoxicity Assays

Target cells were plated at densities of 500 cells/100 µl culture medium per well in 96-well plates. After overnight incubation at 37 °C, 100 µl of culture media containing serial dilutions of ADC were added. After 96 h, 50 µL of PrestoBlue (Thermo Fisher Scientific, Waltham, MA, USA) was added to each well, and the plates were read for fluorescence 30 min later. Data were expressed as % viability compared with that of control untreated cells and calculated EC50 via GraphPad software version 10.4.1.

### 4.4. Mouse Models for the Evaluation of ADC Activities

The Institutional Animal Care and Use Committee at Beth Israel Deaconess Medical Center approved all animal experiments (protocol numbers 100-2011 and 111-2014). Antibodies, either naked or conjugated with a payload, were injected intraperitoneally in all of the experiments performed in the study.

For toxicity, eight-week-old female C57BL/6 mice (Charles River Laboratories, Shrewsbury, MA) were employed, and ADCs were introduced in two different doses (5 or 10 mg/kg) with the dosing scheme of q4dx4 (four-day intervals for four cycles). Each study group contained 10 mice and was repeated twice in side-by-side experiments for all three ADCs (2A7A-LP2, 3m2A7A-LP2, and Ctl-LP2), totaling n = 20/group.

For efficacy, eight-week-old female athymic nude mice (Charles River Laboratories) were employed and dosed with antibodies at 3 mg/kg, either in one single injection or q2dx3 (three cycles with two-day intervals).

Adenoviral vectors, containing either mock control or mouse VEGF-A^164^ (Ad-VEGF-A), were described previously [4,8]. The total number of mice used in each different efficacy study was presented under the corresponding Figure Legend of each figure showing the results of the efficacy studies. Bright-field ear images were taken with a live cam attached to a Wild M400-Photomacroscope (Martin Microscope, Easley, SC, USA). Ears were fixed in paraformaldehyde–glutaraldehyde or in paraformaldehyde alone for electron or light microscopy as described previously [8].

### 4.5. Quantification of Plasma Volumes in Ear Sites

After iv injection, Evans blue dye (EBD) was extracted from biopsy sites and measured spectroscopically to provide a quantitative measure of local plasma albumin content, as described previously [4,25]. The total plasma volume (μL) in each 8 mm biopsy of ear tissue was obtained by conversion of the total EBD amount (ug) from the 8 mm ear extract to the respective mouse’s plasma EBD concentration (μg/μL).

### 4.6. Statistical Analysis

Statistical analysis was performed with GraphPad Prism (La Jolla, CA, USA) software version 10.4.1. Log-rank (Mantel–Cox) test and Student’s *t*-test were applied to assess the significance of differences in survival and local EBD quantity, respectively.

## 5. Conclusions

A TM4SF1-directed ADC (3m2A7A-LP2) effectively targeted the endothelial cells lining angiogenic blood vessels induced by an adenovirus expressing VEGF-A while sparing the normal vasculature in mice in vivo. TM4SF1 was originally discovered as a tumor cell-associated antigen and is expressed by a majority of tumor cells in solid tumors [7]. Our previous preclinical studies using human xenograft tumors in mice have demonstrated better tumor regression outcomes with the combination treatment of a human TM4SF1-directed ADC (V1.1-LP2) targeting human tumor cells and a mouse TM4SF1-directed ADC (2A7A-LP2) targeting tumor vessels than was achieved with either alone [12]. Thus, TM4SF1 is an attractive ADC target potentially enabling pan-solid tumor therapy through dual targeting of tumor blood vessels and tumor cells. The sensitivity of cells to TM4SF1-directed ADCs appears to be determined by TM4SF1 expression levels that surpass a threshold that enables TMED formation for the subsequent internalization of ADCs via microtubules to the nucleus. The details of this novel internalization pathway may be elucidated by future studies in the spatial biology of TMEDs at nano-scale resolution, and the proteomics of TMED composition by mass-spec; such pathway elucidation will enable further improvements to the design and therapeutic efficacy of a newer generation of TM4SF1-directed ADCs.

## Data Availability

The original contributions presented in this study are included in the article/Supplementary Material. Further inquiries can be directed to the corresponding author.

## Supplementary Materials

The following supporting information can be downloaded at: https://www.mdpi.com/article/10.3390/ijms27104437/s1.
