# Supplementary material for: TM4SF1-Directed Antibody–Drug Conjugates Selectively Destroy Newly Formed Blood Vessels Induced by VEGF-A
Source: Int J Mol Sci. 2026 May 15;27(10):4437. doi: 10.3390/ijms27104437 (PMC13207009; doi:10.3390/ijms27104437)
Supplement: Supplementary file 1 [file ijms-27-04437-s001.zip › Supplementary Materials and Methods.pdf]

Supplemental Materials and Methods

Antibodies and antibody-drug conjugates (ADCs).

Three antibodies were employed to generate three ADCs in this study: (i) 2A7A is a rabbit anti-mouse TM4SF1 monoclonal antibody in which the rabbit variable region was engrafted to a human IgG1 constant region [1]; (ii) 3m2A7A is an effector function ablated 2A7A formed by mutating three amino acids (L234A, L235A, and G237A) in the heavy chain to diminish Antibody-Dependent Cellular Cytotoxicity (ADCC) activities [2,3] (**Fig. 1A**); and (iii) 8.8 is a non-targeting isotype-matched control (Ctl) antibody. The linker-payload LP2 (mc-3377) comprises a maleimidocaproyl (mc) linker that is attached via an amide bond to PF-06463377, a Pfizer-developed synthetic analog of dolastatin-10 derived microtubule cytotoxin whose structure resembles that of momomethyl auristatin F (MMAF) [4]. The details of the LP2 conjugation chemistry were reported in our previous studies and generated three ADCs (2A7A-LP2, 3m2A7A-LP2 and Ctl-LP2) [1]. All ADCs were purified by size exclusion chromatography and suspended in PBS, pH 7.4. The mutation did not alter the 3m2A7A binding affinity to its antigen TM4SF1 (data not shown), and 3m2A7A-LP2 ADC routinely produced 1-5 nM EC50 cytotoxic activities in both MS1 (immortalized mouse endothelial cells) and $293^{mTM4SF1}$ (mouse TM4SF1 stably transfected HEK293 cells) cells *in vitro*, comparable to 2A7A-LP2 (**Fig. 1B**).

VEGF-A provoked mouse skin angiogenesis model, and light and electron microscopy

Adenoviral vectors expressing mouse VEGF-A^{164} (Ad- VEGF-A^{164}) and Lac-Z (Ad-Lac-Z) were described in detail in previous studies [5]. Briefly, adenoviruses (5×10^7 plaque forming units in 10 µL of phosphate-buffered saline/3% glycerol) were injected intradermally into the ears of nude mice to induce angiogenesis via a mouse VEGF-A^{164} overexpressing vector (Ad-VEGF-A) or to retain normal vasculature via a mock control vector (Control). Bright field ear images were taken with a live cam attached Wild Photo Mikroskop M400 stereo photomicroscope (Martin Microscope, SC). For electron microscopy, ears were fixed in paraformaldehyde-glutaraldehyde and the tissue was processed as previously described [5]. For histology, tissues were fixed in 4% paraformaldehyde and embedded in paraffin for H&E staining as described in our earlier studies [5].

Quantification of intravascular plasma volumes in adenovirus–injected ear sites

Quantification of Evans blue dye (EBD), a low molecular weight dye that binds to plasma albumin and provides a faithful tracer of intravascular plasma volumes, has been described in detail in our earlier studies [6]. Briefly, mice were anesthetized with isoflurane, ears were photographed, and EBD (50 uL/20 g mouse of 1.0% EBD in saline) was introduced via retro-orbital injection of the venous sinus. Eight minutes later, at a time that allowed EBD equilibration in blood and filling of the angiogenic vasculature, ears were photographed again, and 100 uL of blood was collected by retro-orbital puncture into a heparin tube. Mice were then euthanized for harvest of an 8-mm biopsy punch that encompasses the entire region of ear angiogenesis into 0.5 mL formamide. Plasma was gathered from the blood via centrifugation (15,000 × *g* for 10 minutes) and tissue EBD was extracted at 56°C incubator for 3 days. The EBD concentration (µg/mL) in each sample was determined with absorbance at 620 nm via a standard curve. The volume of plasma (µL) in each ear biopsy tissue was obtained by conversion of the total EBD amount from the tissue extract with the EBD concentration in the plasma (µg/µL) of the respective mouse.

References:

1. Visintin A, Knowlton K, Tyminski E, Lin CI, Zheng X, Marquette K, Jain S, Tchistiakova L, Li D, O'Donnell CJ, Maderna A, Cao X, Dunn R, Snyder WB, Abraham AK, Leal M, Shetty S, Barry A, Zawel L, Coyle AJ, Dvorak HF, Jaminet SC (2015) Novel Anti-TM4SF1 Antibody-Drug Conjugates with Activity against

Tumor Cells and Tumor Vasculature. Mol Cancer Ther 14 (8):1868-1876. doi:10.1158/1535-7163.MCT-15-0188
2. Schlothauer T, Herter S, Koller CF, Grau-Richards S, Steinhart V, Spick C, Kubbies M, Klein C, Umana P, Mossner E (2016) Novel human IgG1 and IgG4 Fc-engineered antibodies with completely abolished immune effector functions. Protein engineering, design & selection : PEDS 29 (10):457-466. doi:10.1093/protein/gzw040
3. Hale G, De Vos J, Davy AD, Sandra K, Wilkinson I (2024) Systematic analysis of Fc mutations designed to reduce binding to Fc-gamma receptors. mAbs 16 (1):2402701. doi:10.1080/19420862.2024.2402701
4. Maderna A, Leverett CA (2015) Recent advances in the development of new auristatins: structural modifications and application in antibody drug conjugates. Molecular pharmaceutics 12 (6):1798-1812. doi:10.1021/mp500762u
5. Shih SC, Zukauskas A, Li D, Liu G, Ang LH, Nagy JA, Brown LF, Dvorak HF (2009) The L6 protein TM4SF1 is critical for endothelial cell function and tumor angiogenesis. Cancer Res 69 (8):3272-3277. doi:10.1158/0008-5472.CAN-08-4886
6. Sitohy B, Chang S, Sciuto TE, Masse E, Shen M, Kang PM, Jaminet SC, Benjamin LE, Bhatt RS, Dvorak AM, Nagy JA, Dvorak HF (2017) Early Actions of Anti-Vascular Endothelial Growth Factor/Vascular Endothelial Growth Factor Receptor Drugs on Angiogenic Blood Vessels. Am J Pathol 187 (10):2337-2347. doi:10.1016/j.ajpath.2017.06.010
