# Supplementary material for: TM4SF1-Directed Antibody–Drug Conjugates Selectively Destroy Newly Formed Blood Vessels Induced by VEGF-A
Source: Int J Mol Sci. 2026 May 15;27(10):4437. doi: 10.3390/ijms27104437 (PMC13207009; doi:10.3390/ijms27104437)
Supplement: Supplementary file 1 [file ijms-27-04437-s001.zip › TM4SF1-ADC paper; Supplementary Figures.pdf]

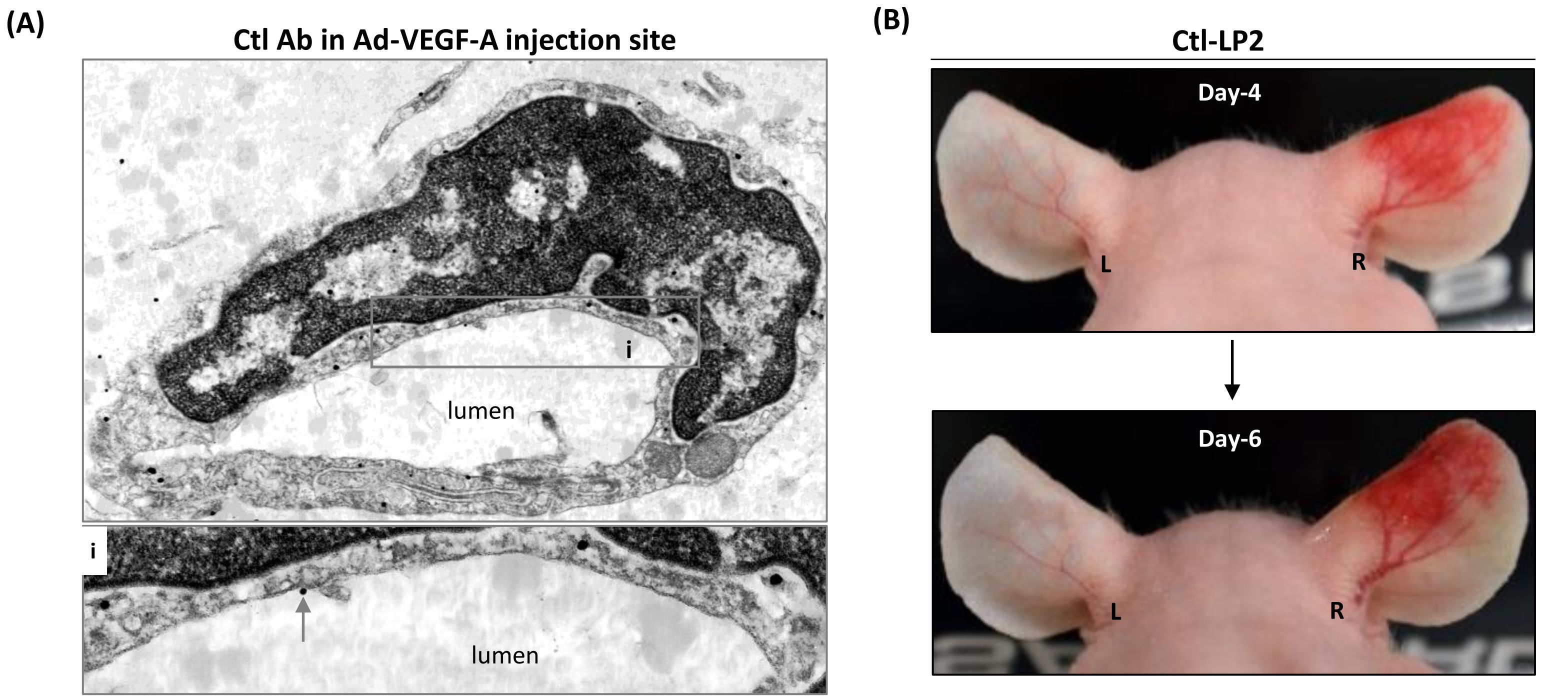

Figure S1. Isotype matched control antibody activities in Ad-VEGF injected ear. Nude mice received intradermal ear injections of 2.5x10^{7} pfu mock control (Control, left-ear L) or mouse VEGF-A^{164} (Ad-VEGF-A, right-ear R) adenovirus. Four days later, mice received a single 3 mg/kg intraperitoneal injection of (**A**) Ctl antibody or (**B**) Ctl-LP2. Representative images from three different ears shown. (**A**) Homing of Ctl antibody to microvasculature in Ad-VEGF injected sites. Mouse ears were harvested for immuno-electron microscopy 6-hour later. Nanogold-labeled anti-human IgGs were used to locate Ctl antibody. Minimal gold particles (grey arrow) on microvascular endothelium at Ad-VEGF injected sites. (**B**) Ctl-LP2 effect on Ad-VEGF injected ear. Ear images show no apparent effect of Ctl-LP2 on either the Ad-VEGF-A (R) or mock control (L) ears 48-hours after the injection.

VEGF-A–induced tumor surrogate model

A	B	C
**Days**<br>(after subdermal ear injections of VEGF-A adenovirus)	**Sequential vessel development and maturation**	**Experimental Plans**
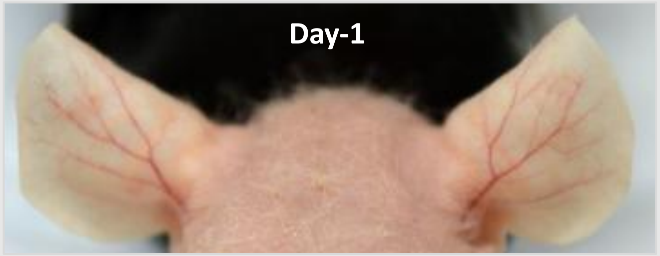	**Stage 1:**<br>• **Capillaries (Cp)**<br>• **Feeder arteries (FA)**<br>• **Draining veins (DV)**	**Plan A**<br>(Day-1 ADC injection)
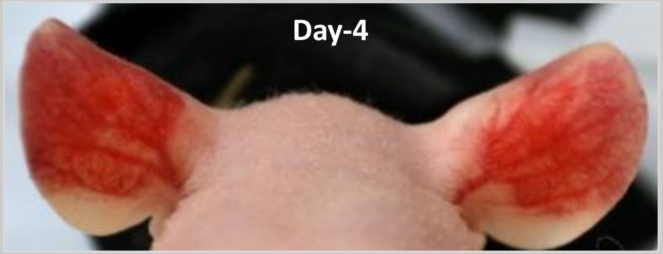	**Stage 2:**<br>• **Mother vessels (MV)**<br>• **Capillaries (Cp)**<br>• **Feeder arteries (FA)**<br>• **Draining veins (DV)**	**Plan B**<br>(Day-4 ADC injection)
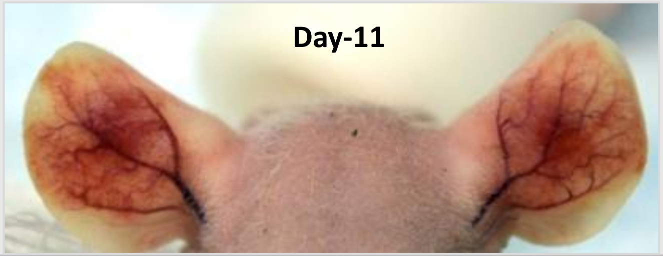	**Stage 3:**<br>• **Glomeruloid microvascular proliferations (GMP)**<br>• **Capillaries (Cp)**<br>• **Feeder arteries (FA)**<br>• **Draining veins (DV)**	**Plan C**<br>(Day-11 ADC injection)
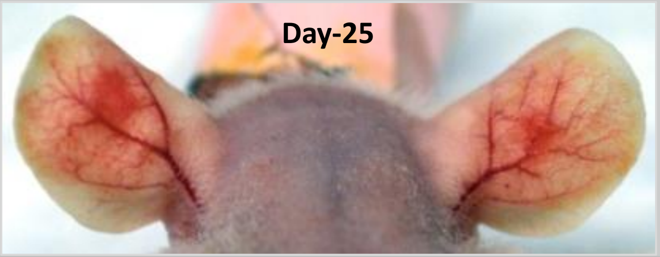	**Stage 4:**<br>• **Vascular malformations (VM)**<br>• **Feeder arteries (FA)**<br>• **Draining veins (DV)**	**Plan D**<br>(Day-25 ADC injection)

Figure S2. Sequential pattern of new blood vessel development in nude mice ears following a single injection of 2.5×10^7 pfu Ad-VEGF-A. Intradermal injection of adenovirus containing mouse VEGF-A^{164} overexpression plasmid progressively induced six different sub-types of blood vessels over the course of 25 days. **Column-A:** representative ear images from Day-1, -4, -11 and -25 after the Ad-VEGF-A^{164} injection to signify four critical blood vessel development stages. **Column-B:** the evolution of six different types of blood vessels during 25 days of observation. **Column-C:** the four experimental plans selected to examine anti-TM4SF1 ADC effects on different stages of vessel development.

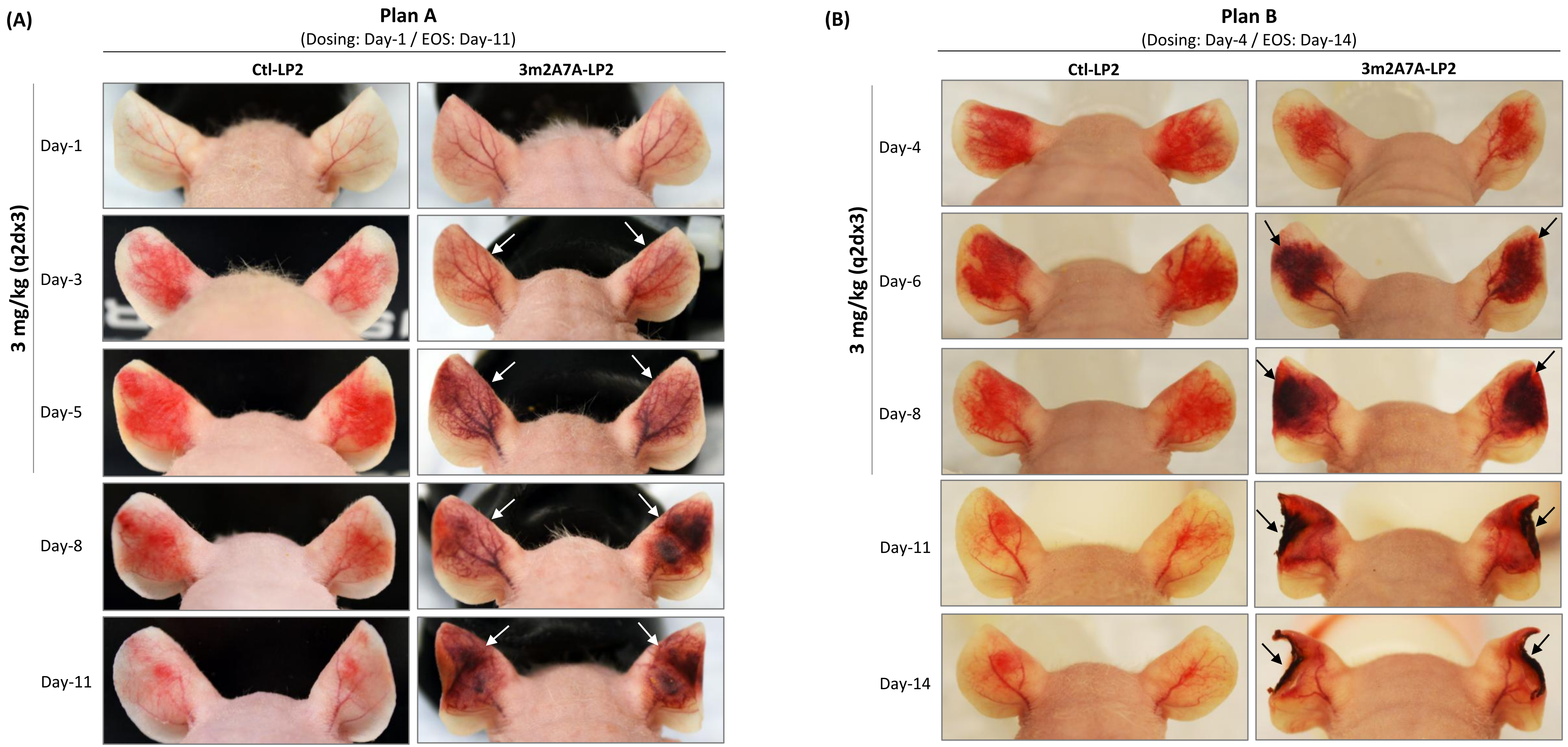

Figure S3. An ADC directed against murine TM4SF1 3m2A7A-LP2 specifically eliminates Ad-VEGF-A-induced angiogenic blood vessels. The experimental plan is described in the legend to Figure 4. Images were taken immediately after the dosing of each ADC (Ctl-LP2 or 3m2A7A-LP2; 3 mg/kg, q2dx3) and every three days thereafter throughout a ten-day experimental period. In comparison to mice treated with Ctl-LP2, mice treated with 3m2A7A-LP2 experienced targeting of angiogenic vessels (indicated by arrows) after the first dosing in Plan-A (**A**) and in Plan-B (**B**), and after the second dosing in Plan-C (**C**). Plan-D showed insignificant differences between treatments (**D**). EOS: end of study.

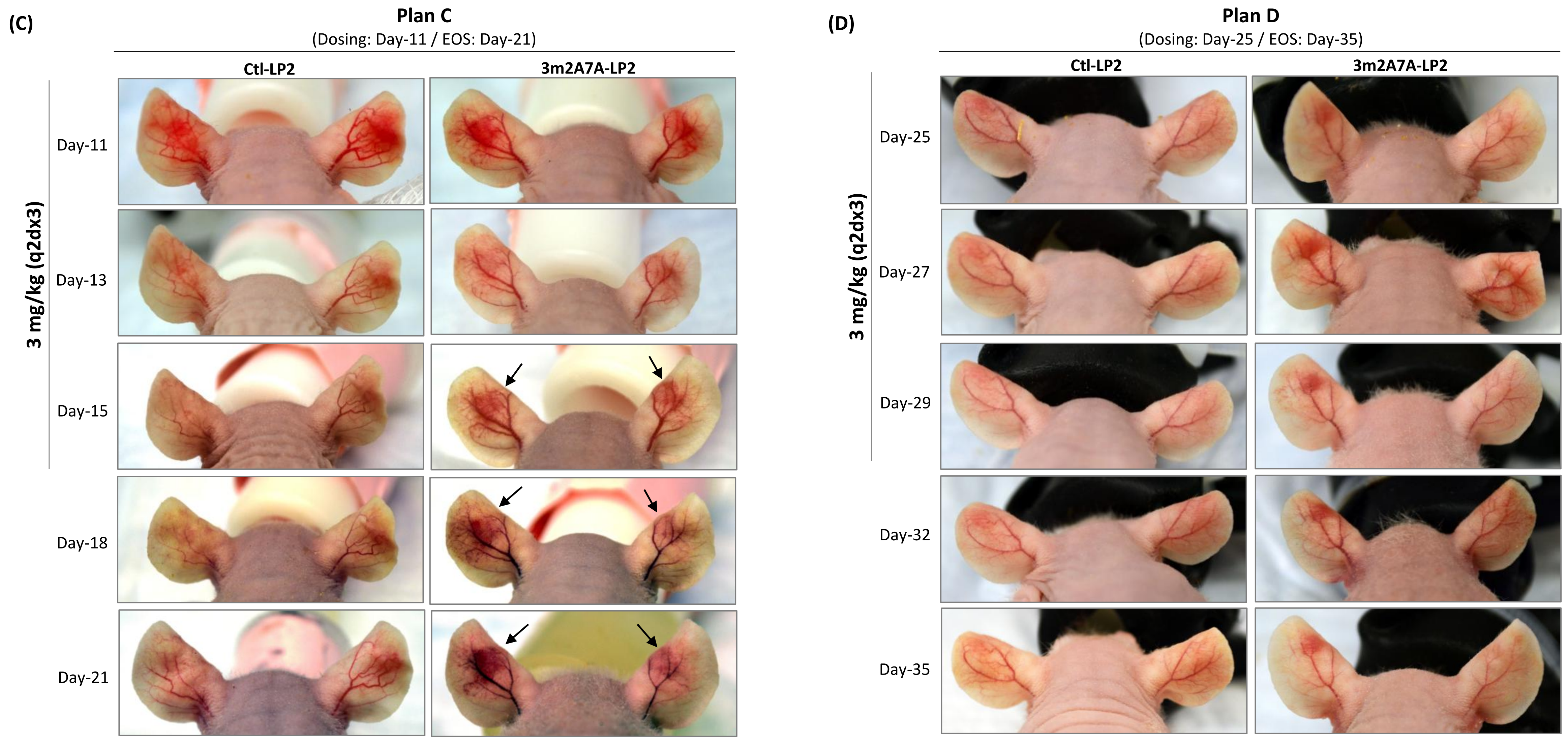
(C)
Plan C
(Dosing: Day-11 / EOS: Day-21)
Ctl-LP2
3m2A7A-LP2
3 mg/kg (q2dx3)
Day-11
Day-13
Day-15
Day-18
Day-21
(D)
Plan D
(Dosing: Day-25 / EOS: Day-35)
Ctl-LP2
3m2A7A-LP2
3 mg/kg (q2dx3)
Day-25
Day-27
Day-29
Day-32
Day-35
